# The Effects of Air Pollution, Sea Exposure and Altitude on COVID-19 Hospitalization Rates in Italy

**DOI:** 10.3390/ijerph18020452

**Published:** 2021-01-08

**Authors:** Ennio Cascetta, Ilaria Henke, Luigi Di Francesco

**Affiliations:** 1 Department of Civil, Construction and Environmental Engineering, University of Naples “Federico II”, 80125 Napoli, Italy; ennio.cascetta@unina.it; 2 Department of Engineering, University of Campania “Luigi Vanvitelli”, 81031 Aversa, Italy; luigi.difrancesco13@gmail.com

**Keywords:** COVID-19, air pollution, PM_2.5_, human health, sea air exposure, regression model, high altitude

## Abstract

Early known cases of COVID-19 emerged in late 2019 in the city of Wuhan (China) and in a relatively short time, it has reached more than 200 countries up to July 2020. In Italy, from 21 February 2020, (first official Italian positive case of COVID-19) until 27 July 2020, 246,286 confirmed cases were observed of which over 68,150 (28%) needed hospitalization and 35,112 died. In recent scientific research, it has been shown that the severity of symptoms and mortality rates were different not only among the various countries of the world but also in different regions of the same country. This research investigates whether and by how much air environmental conditions (such as exposure to fine particulate matter-PM_2.5_, sea air masses and altitude) influences the risk of hospitalization due to COVID-19 in Italy, once the spreading of the virus and the percentage of the elderly in the population have been accounted for. A log-linear multiple regression model was estimated where the log of the ratio of hospitalized patients per inhabitant, since the beginning of the epidemic up to July 27, has been considered as a dependent variable. Among the independent variables, the ones that have been taken into account are the spreading of the virus, the rate of people over 50 years of age, the concentration of PM_2.5_, the rate of population living by the sea, the rate of green public space for each resident and the ratio of population living at a high altitude. The results showed an increase in the hospitalization rate in terms of the percentage of people over 50 and the average concentration of PM_2.5_. If average limits of PM_2.5_ concentration allowed by the current European regulations (25 µg/m^3^) were respected in all Italian provinces, that would have led to 7339 less hospitalizations for COVID-19 (−11%). On the contrary, near the coast there were lower hospitalized cases in the referred period. In the hypothetical case that no Italians lived near the sea, about 1363 (+2%) more hospitalizations would have been recorded in the analysis period in addition to the effect of a lower PM concentration. This paper wanted to investigate which are the areas with a higher risk of hospitalization in Italy, so as to help the Italian Government to strengthen Health System measures, predicting the most suffering areas and health care systems. According to the results, this is directly related to the severity of symptoms which decreased with the long-time exposure to the sea.

## 1. Introduction

Severe acute respiratory syndrome coronavirus 2 (SARS-CoV-2) is the pathogen of the COVID-19 disease [1]. Early cases of the virus emerged in China, and since December 2019, Chinese health authorities have drawn international attention on a cluster of pneumonia cases in the city of Wuhan. On 9 January 2020, the pathogen causing viral pneumonia among affected individuals has been recognized as the SARS-CoV-2, later called COVID-19. In March 2020, the World Health Organization (WHO) formally declared the COVID-19 outbreak as a global pandemic. At the end of July, a total of 16.3 million cases and 650,000 deaths have been confirmed worldwide. Italy was the first country after China to be hit hard by the epidemic. On 21 February 2020, the COVID-19 pandemic started in Italy principally in the north (Lombardy was the most affected region). From 21 February 2020, until 27 July 2020, 246,286 confirmed cases were observed among which over 68,150 needed hospitalization and 35,112 died. In July 2020, significant differences were observed in the various countries of the world, both for the spreading rate and for the mortality rate from COVID-19 [2]. The mortality rate was different, not only between the various countries of the world but also in different regions of the same country [2]. One of the principal severe diseases caused by COVID-19 is linked to progressive respiratory failure due to massive alveolar damage, leading to probable death [3]. Numerous epidemiological studies have highlighted the link between the air pollution and hospital admissions for a variety of different health reasons including a number of respiratory diseases [4,5,6,7,8]. Mostly short-term exposure to fine particulate matter (PM_2.5_) increases the risk for hospital admission for cardiovascular and respiratory diseases [9]. There is also solid scientific literature describing the role of atmospheric particulate matter as an effective carrier, i.e., transport and diffusion vector for various biological and chemical contaminants, and therefore also for viruses [10,11,12]. However, the debate is still open on this issue. Indeed, Bontemi [13] comparing the spread of COVID-19 and the value of the PM between two Italian provinces conclude that is not possible to assert that COVID-19 diffusion mechanism also occurs through the air, by using PM_10_ as a carrier.

Starting from these considerations, recent studies showed a connection between air pollution and more severe prognoses for COVID-19. An American study showed how the increase in mortality rate was a function of a slight increase in prolonged exposure to PM_2.5_ [14]. Similar results were obtained in Italy [15,16]; it has been demonstrated that exposure to air pollution could increase the vulnerability of patients with COVID-19 and that it could make their prognosis worse.

It has also been shown that sea air masses give an important contribution to reduce PM concentration levels in coastal areas [17,18]. In the study conducted in China, has proven that the city of Ningbo is managed by continental and maritime air masses and has shown how in the maritime air masses there is a lower concentration of PM_2.5_ which increases in continental air flows [18]. Medical literature shows that living near the coast can increase health and wellbeing [17,18,19,20]. A recent study carried out by the University of Gand (Belgium) and the Flamine Marine Institute (VLIZ) has found that seawater contains microbiota and biogenic molecules which could affect human health [21]. It has also been shown that living in coastal areas improves vitamin D concentration helping to reduce cardiovascular diseases [22,23,24,25]. In addition to the areas near the coast, the mountainous areas are those where there are on average lower concentrations of pollution with a negative correlation between PM_2.5_ and the elevation [18,26,27]. The scientific literature about the health effect of the altitude on the residents has provided conflicting results. COVID-19 cases have been reported from high-altitude regions of Europe, Asia, South America, North America, and Africa [28,29,30,31,32]. Arias-Reyes et al. [29] suggested that physiological acclimatization/adaptation in Tibet and high-altitude regions of Bolivia and Ecuador, may have protective effects on SARS-CoV-2 virus. According to this assumption, Xi et al. [31] have the same results on the Qinghai-Tibetan plateau, China. Quevedo-Ramirez et al. [33] also confirmed that COVID-19 infection is reduced at high altitude, while case-fatality rate was not, according to the studies of Segovia-Juarez et al. [34]. However, according Pun et al. [35] these results should be carefully interpreted, indeed, there is little supporting evidence for any protective benefit to high-altitude hypoxia. Hence, a study conducted by Woolcott and Bergman [36] suggested higher mortality rates in U.S. counties located at ≥2000 m elevation against those located <1500 m (12.3 vs. 3.2 per 100,000; *p* < 0.001). The same was found in Mexico, among men the risk of death was 31% higher at ≥2000 m versus that at <1500 m while no association was found among women or subjects 65 years of age and older. Specific to the Italian case study, several studies analyzed the effect due to the COVID-19 pandemic (e.g., [13,15,16,37,38]), but the most of them did not analyze the effects that influenced the severity of symptoms considering the hospitalization. Starting from these considerations, the aim of this study is to evaluate if (and by how much) air quality (as exposure to fine particulate matter-PM_2.5_, to sea air masses and altitude) could influence the COVID-19 effects in terms of its hospitalization rate, given the diffusion of the virus (ratio of the positive test and the number of tests carried out). To the best of authors’ knowledge, this paper is the first to evaluate the impact of sea exposure on the severity of symptoms due to COVID-19. In addition, for the first time in Italy, the effect of the PM exposures, sea proximity and the high altitude on the hospitalization rate was estimated.

## 2. The Case Study and the Methodology Proposed

Data from 107 Italian provinces and 20 Italian regions were collected up to 27 July 2020. The Civil Protection in Italy provides data about number of positive cases for COVID-19 (number of infected cases), number of tests at the provincial level and number of dead and recovered patients at the regional level. In Italy, the Health System is public, and in the period of health emergency studied, there were no cases of patients who needed hospitalization and were not hospitalized. The number of hospitalized patients is provided only at the regional level, so it was necessary to estimate the number of hospitalized residents for each province according this formulation:

(1) $$\mathrm{Number}\;\mathrm{of}\;\mathrm{hospitalized}\;{\mathrm{residents}}_{i}=\mathrm{Number}\;\mathrm{of}\;\mathrm{hospitalized}\;{\mathrm{residents}}_{k}*\frac{Num.Positive\;test_{i}}{Num.Positive\;test_{k}}$$

where:

*Number of hospitalized residents_i_* is the total number of inhabitants resident in the provincial *i* belonging to the region *k* which were hospitalized for COVID-19 from March to July 2020;

*Number of hospitalized residents_k_* is the total number of inhabitants resident in the region *k* which were hospitalized for COVID-19 from March to July 2020. These data were taken from the Civil Protection;

$Num.Positive\;test_{i}$ is the total number of positive tests of COVID-19 for residents in the provincial *i* belonging to the region *k* from March to July 2020;

$Num.Positive\;test_{k}$ is the total number of positive tests of COVID-19 for residents in the region *k* from March to July 2020.

**Figure 1:**
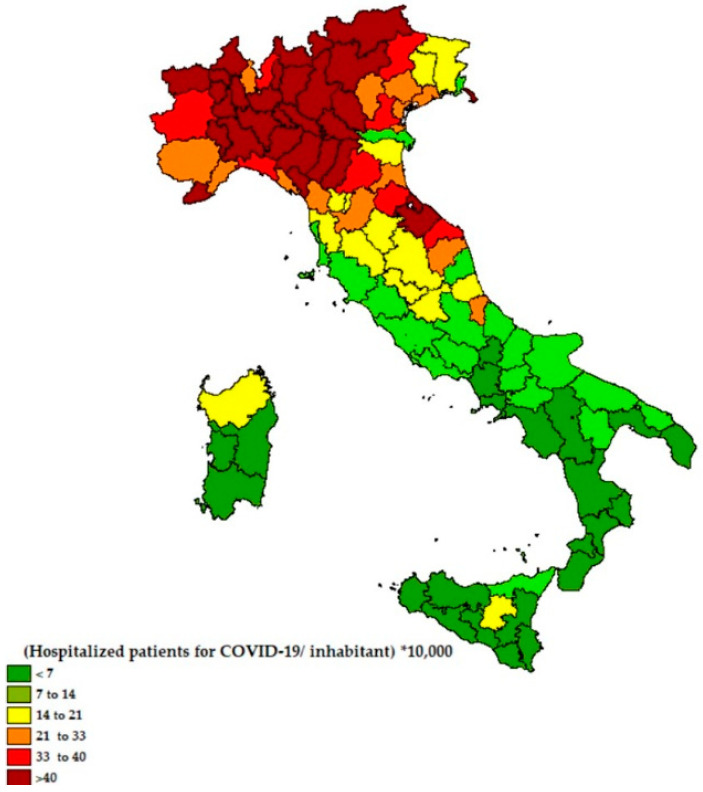
The ratio of hospitalized patients per inhabitant for each Italian province since the beginning of the epidemic to 27 July (number of people hospitalized for COVID-19/resident ∗ 10,000).

The Figure 1 shows the ratio of hospitalized patients for each Italian province since the beginning of the epidemic to 27 July 2020.

In this research, the levels of pollution were also analyzed (specifically the PM_2.5_ e PM_10_, those most harmful on human health) as the annual average pollution level of each of the Italian provinces. These data were estimated by the Regional Environmental Protection Agencies (Agenzie Regionali per la Protezione Ambientale, ARPA).

**Figure 2:**
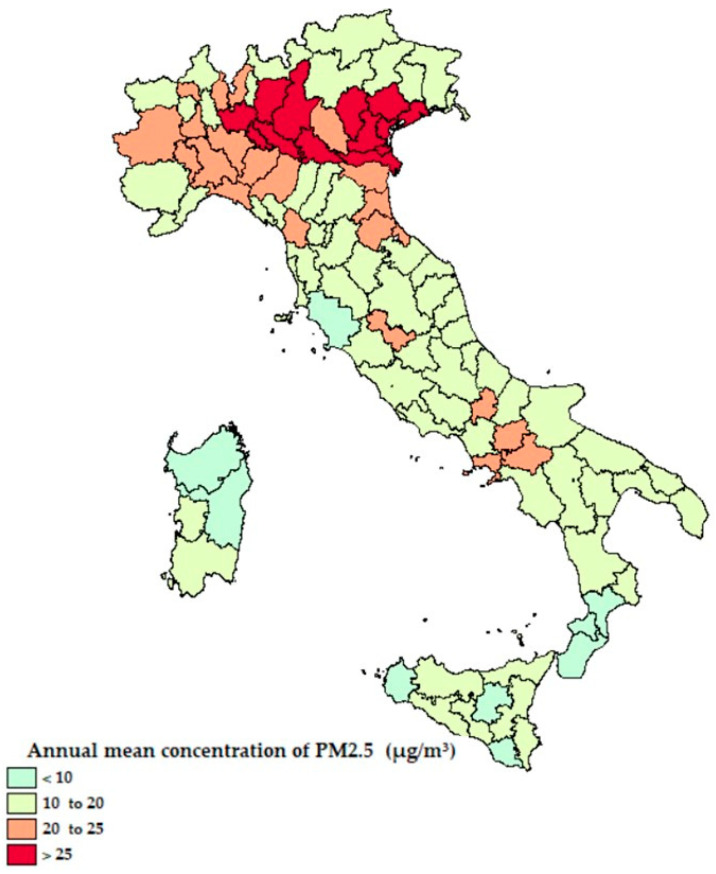
The annual mean concentration of fine particulate matter (PM_2.5_) for each Italian province.

**Figure 3:**
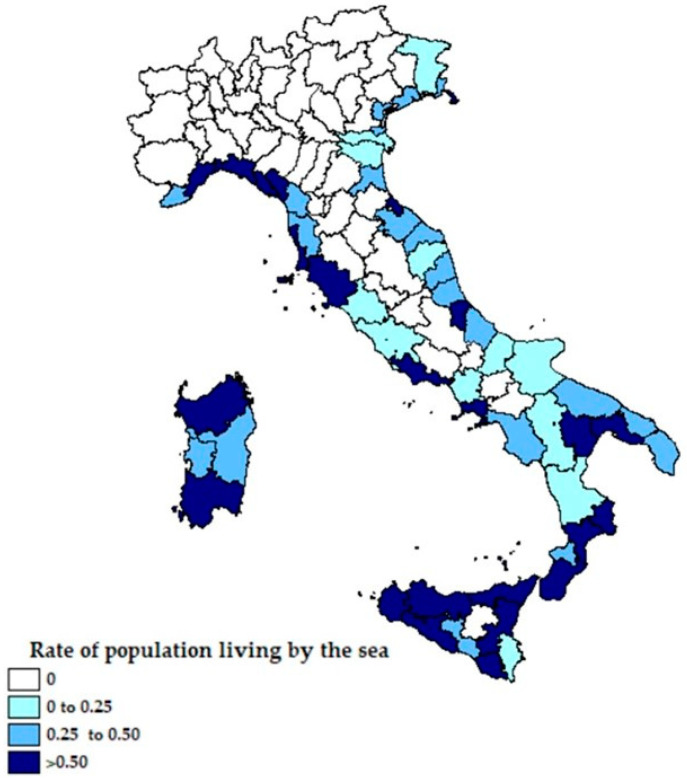
Rate of population living by the sea for each Italian province.

**Figure 4:**
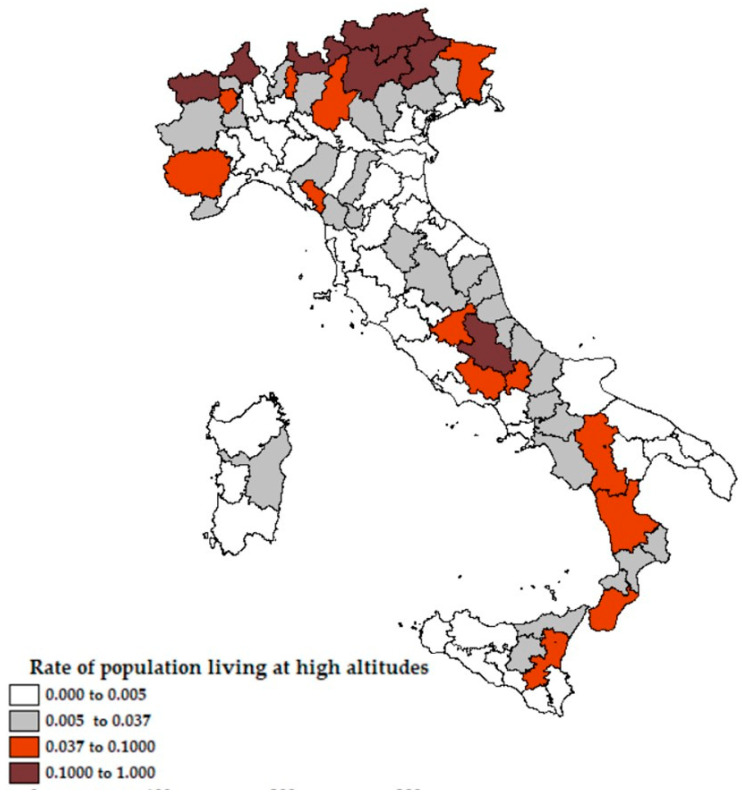
Rate of population living at high altitudes (1200 m above sea level) for each Italian province.

From Figure 1 and Figure 2 it emerges that the higher ratio of hospitalized patients with COVID-19 was recorded in Northern Italy, areas with more emissions of PM_2.5_ and no exposure to the sea, while the ratio is lower in the areas closest to the coast. In Figure 3, the percentage of the population living by the sea has been reported and in Figure 4 the percentage of the population who lives above 1200 m for each Italian province. Comparing the maps of these two figures, most of the people in Italy live near the coast (22% of the Italian population compared to 2% of the Italian population living at high altitudes).

Starting from these considerations, in order to be able to estimate if the weight of air pollution exposure, sea proximity and altitude could influence the severity of symptoms of COVID-19, different regression models were tested evaluating the statistical significance of several zone-specific attributes.

Overall, several models with functional transformation of the variables and different combinations of them were estimated. Considering the dependent and independent variable combinations, a linear combination (linear regression), a logarithmic combination (logistic regression) and a combination of them (log-linear regression) were performed. Starting from the parameter estimation, only those deriving from the log-linear regression were considered representative, so this paper focused on this one.

In the model formulation tested, it was considered as a dependent variable of the Natural logarithm of the number of hospitalized residents of the province *i* for COVID-19 since the beginning of the epidemic (21 February) up to 27 July, divided into the total number of residents of the province *i* ∗ 10,000.

The independent variables introduced for each province have been grouped into three macro-categories relative to: (i) the COVID-19 spread, (ii) the air pollutants, (iii) the geographical conditions.

To take into account different numbers of COVID-19 positive tests in different provinces in the Italian provinces (first group), the ratio of the number of positive tests and the total number of tests carried out was considered.

The second group’s variables concerned air pollutants, regarding PM_10_ and PM_2.5_. Indeed, considering the complexity due to the big amount of air pollutants, in this analysis we chose to focus on those with harmful effects on human health in terms of chronic health effects (that are consistent with [39,40]). So, in this analysis the average annual values of daily concentration (µg/m^3^) of PM_2.5_ and PM_10_ particles were considered as independent variables.

Finally, the geographical variables have been tested to investigate if the geographic features such as sea air masses and altitude could influence the risk of hospitalization for COVID-19 in Italy. Therefore, the following variables have been considered: the length of the sea coast/the ratio between the length of the coast and the province’s surface/the ratio between the number of residents who live near the sea and the number of residents; the proportion of the population living at an altitude greater than or equal to 600 m above sea level/the ratio between the population living at an altitude above 600, 900 and 1200 m and the number of residents; the average yearly temperature for each province/the average temperature observing during the first quarter of 2020 for each province.

In addition, as suggested by Coker et al. [39], several control variables have been introduced that may plausibly have effects on the hospitalization rate for each province, including population density/average age of population/ratio between the number of residents older than 40, 50 and 60 years old and the amount of residents/percentage of women/percentage of men/ratio between the public green space and the residents on each province/ratio between workers and residents.

Some data used in this study were not available at the provincial level, so, applying formula 1, they were estimates from regional available data.

In order to be able to estimate the impact that air pollution exposure and sea proximity could have on the severity of symptoms of COVID-19, as measured by the hospitalization rate, once other structural variables such as the diffusion of COVID-19 epidemic and the age composition of the population were accounted for, the log-linear regression Model 1 was estimated:

*LN (hospitalization rate)_i_* = *β*_0_ + *β*_1_ ∗ *DIFFUSION_i_* + *β*_2_ ∗ *POP.OVER 50 RATE_i_* + *β*_3_ ∗ *PM_2.5i_* + *β*_4_ ∗ *SEA EXPRE RATE_i_* + *β*_5_ ∗ *GREEN RATE_i_* (2)

where:

*LN (hospitalization rate)_i_* is the Natural logarithm of the number of hospitalized residents of the province *i* due to COVID-19 since the beginning of the epidemic (February 21) to July 27, divided to the total number of the province residents *i* ∗ 10 thousand. The number of residents hospitalized for each province was estimated according to the formulation (1);

*β*_0_ is the constant specific of the alternative measuring all the attributes not explicitly considered;

*COVID DIFFUSION_i_* is the ratio of the number of positive tests for resident in provincial *i* and the total number of tests carried out in the province *i* since the beginning of the epidemic (21 February) to 27 July. This variable represents the cumulated spreading of the virus;

*POP.OVER 50 RATE_i_* is the ratio between the number of residents older than 50 years old and the number of residents living in a province *i;*

*PM_2.5i_* is the average annual value of daily concentration (µg/m^3^) of PM_2.5_ particles in the province, estimated from Regional Environmental Protection Agencies (Agenzie Regionali per la Protezione Ambientale, ARPA);

*SEA EXPRE RATE_i_* is the ratio between the number of residents living near the sea and those living in the province *i*. Due to the absence of more detailed data, the number of people who may benefit from sea exposure was estimated by the sum of the coastal cities’ populations;

*GREEN RATE_i_* is ratio between the public green space and the residents in each province *i*. It was expressed in square meters per inhabitant.

The second model performed, Model 2, is explained as follows:

*LN (hospitalization rate)_i_* = *β*_0_ + *β*_1_ ∗ *COVID DIFFUSION_i_* + *β*_2_ ∗ *POP.OVER 50 RATE_i_* + *β*_3_ ∗ *PM2.5_i_* + *β*_4_ ∗ *SEA EXPRE RATE_i_* + *β*_5_ ∗ *GREEN RATE_i_* + *β*_6_ ∗ *ALTITUDE RATE_i_* (3)

This differs from the Model 1 by the addition of one variable:

*ALTITUDE RATE_i_* is the ratio between the number of residents who live at high altitude (1200 m above sea level) and the number of residents who live in the province *i.*

For each municipality, the number of inhabitants divided by the altitude ranges is provided by the ISTAT website, while *β*_0_, *β*_1_, *β*_2_, *β*_3_, *β*_4_, *β*_5_ and *β*_6_ are the parameters estimated by the model.

## 3. Results and Discussion

The tables below (Table 1 and Table 2) show the main estimation results. In Table 1, shows the model parameters, while the main results are reported in Table 2. The ratio of hospitalized patients per inhabitant increases with the spreading of the virus (the ratio between the positive tests and all those carried out in the province) and with a higher average age (over 50 years old) of the province. This statement is in accordance with the study of Garg et al. [41], that showed an increasing rate of hospitalization for COVID-19 with a higher average age: among 1482 patients hospitalized, 74% were aged ≥50 years old.

Moreover, another important statement is about the increase in the hospitalization rate with a higher average concentration of PM_2.5_, that is consistent with the American study of Wu et al. [14] which showed the relationship between a long-term exposure to PM_2.5_ and the COVID-19 death rates. In addition, the American study demonstrated that the COVID-19 mortality rate is higher in the regions where there was a long-term exposure to PM_2.5_. This result was confirmed also in Italy (e.g., [16,39]) suggesting an increase in mortality rate with a higher exposure to air pollution. The calibrated model for the Italian case study showed that the PM_2.5_ concentrations explained about 21% of the hospitalization rate. If the average quantitative limits of PM_2.5_ allowed by the current EU regulations (25 µg/m^3^) had been respected in all Italian provinces (reduction in Italy of 4% of emissions, about 1 µg/m^3^), there would be about 7339 less COVID-19 hospitalizations (−11%).

According to the medical literature about the benefits of the sea, the Model 1 was performed to show if this statement could be confirmed in this case. The results showed a small but statistically significant effect of sea exposure that could decrease the severity of COVID-19 symptoms by about 2% in addition to the effect on PM concentrations. So, in the case of no Italians living near the sea, considering the same levels of PM concentration, there would be about 1363 (2%) more hospitalizations in the period analyzed.

Finally, to analyze the effects of high altitudes on the hospitalization rate, the Model 2 was estimated. Despite the altitude having a positive effect (the higher the percentage of people living above 1200 m, the higher the COVID-19 hospitalization rates), it was not statistically significant. This result is consistent to Ezzati et al. [42] that showed that living at higher altitude has no additional effect on the human health.

However, these results clash with the assumptions of several studies, such as Arias-Reyes et al. [29], Xi et al. [31], Quevedo-Ramirez et al. [33], and Segovia-Juarez et al. [33] which suggested a lower incidence of COVID-19 because of a weaker transmission of the virus among high-altitude populations. However, the result obtained in this paper for the Italian case could be explained considering the very low fraction of citizens living above 1200 m in Italy.

In Table 2 the estimation results are given. All the parameters are statistically significant except for that related to the Altitude Rate (Table 1). The sign of the parameters shows that only the variable connected to the sea exposure (*β*_4_) and the public green space (*β*_5_) produced a decrease in the hospitalization rate, with a negative effect. All the other variables, if statistically significant, increased the hospitalization rate (with a positive effect). The significance of these parameters was considered through the use of statistical tests, i.e., standard error and ratio-t reported in Table 1.

The following results are not general and are only valid under specific assumptions of this Italian case study at the analyzed time period, considering the public health emergency system, the proportion of the population living at high altitude and near the sea, the big percentage of the elderly population, the initial spread constant in all the region and so on. Other methods may be able to solve the estimation problems that arise in the optimal least squares (OLS) approach, such as regression discount design (RDD) and they should be investigated in further studies. However, the authors are aware of the phenomenon and of the cause–effect association complexity, so they can only address part of the problem. Indeed, this paper does not take into account the different ways the pandemic has spread for each region and its dynamics. The main investigation should be on: (1) the dynamic of the spreading; (2) the effects on the COVID-19 spreading of the lockdown which are “asymmetric”. Moreover, to validate these results, other case studies should be checked.

## 4. Conclusions

There is strong evidence that environmental pollution damages human health. In particular, the level of air pollution influences symptoms and mortality associated with various respiratory diseases. This research focused its attention on whether and how much environmental air conditions (such as exposure to fine particulate matter PM_2.5_, to sea air and altitude) could influence the risk of hospitalization due to COVID-19 in Italy, considering the diffusion of the virus and the percentage of the elderly in the population. The estimation results showed that, for the Italian case study, the hospitalization rate increased with the percentage of people over 50 years old, as well as with the higher average concentration of PM_2.5_ and decreased with a higher population living by the sea. This means that provinces near the sea, in addition to having benefits on pollution (sea breeze reduces PM concentrations) have a small but statistically significant effect on the aggression of COVID-19, decreasing the hospitalization ratio by about 2%. The altitude, in these performed models, has no significant effect on the level of aggression of COVID-19, in addition to the benefits in PM reductions.

Currently, a second wave is being recorded, so it is even more important to understand which parameters are significant. This paper wanted to determine the areas with a higher risk of hospitalization in Italy, so as to help the Italian Government to strengthen Health System measures. In fact, by knowing where the greater hospitalization rates are it, it can therefore be possible to predict which are the areas where health care systems should be reinforced. According to the results, this is directly related to the severity of symptoms which decreased with the long-time exposure to the sea. So, due to the results, an Italian Government strategy could be successful in the detection of the areas with a greater hospitalization rate, reinforcing the health care systems to prepared for new cases. This could even help in preventing another lockdown, which would be devasting in Italy for economic and social reasons.

## Figures and Tables

**Table 1:** Model parameters.

Variables	Min	Avg	Max
COVID Diffusion (0, 1)	+0.049	+0.144	+0.530
Pop. over 50 rate (0, 1)	+0.346	+0.394	+0.435
PM2.5 (µg/m^3^)	+6	+17.538	+30
Sea Expre Rate (0, 1)	+0	+0.241	+0.961
Green Rate (mq/citizen)	+1.19	+68.28	+667.05
Altitude Rate (0, 1)	+0.0	+0.037	+0.570

**Table 2:** Model estimation results.

	Model 1			Model 2		
	Estimation	Non Robust Standard Error	*t*- Value	Estimation	Non Robust Standard Error	*t*- Value
*β*_0_	−0.775	0.308	−2.515	−0.859	0.333	−2.580
*β*_1_	6.987	0.785	8.900	6.754	0.784	8.619
*β*_2_	2.777	1.013	2.742	2.492	0.931	2.676
*β*_3_	0.0471	0.009	5.284	0.055	0.010	5.734
*β*_4_	−0.168	0.063	−2.669	−0.026	0.012	−2.200
*β*_5_	−0.0005	0.000	−2.746	−0.0004	0.000	−2.704
*β*_6_				1.2556	0.694	1.81
Residual Sum of Squares	22.626			21.628		
Adj. R-Squared	0.646			0.657		
F-statistic	29.088 on 5 and 72			25.552 on 6 and 71		

## Data Availability

Data is contained within the article.
